# Application of Biophysical Techniques to Cellular and Molecular Oncology

**DOI:** 10.3390/cancers15112919

**Published:** 2023-05-26

**Authors:** Diane S. Lidke, Jennifer M. Gillette, Alessandra Cambi

**Affiliations:** 1 Department of Pathology and Comprehensive Cancer Center, School of Medicine, University of New Mexico, Albuquerque, NM 87102, USA; jgillette@salud.unm.edu; 2 Department of Medical BioSciences, Radboud University Medical Center, 6500 HB Nijmegen, The Netherlands; alessandra.cambi@radboudumc.nl

Dysregulated cellular processes drive malignant transformation, tumor progression, and metastasis, and affect responses to therapies. While much is known about the biochemical and genetic drivers of these processes, less is understood about the influence of biophysical properties on oncogenesis. This Special Issue of *Cancers* highlights interdisciplinary research that applies physical science principles and approaches to the study of cancer biology.

The research articles presented in this issue address a broad range of both basic and translational cancer biology problems. A central theme at the crossroads of tumor biology and biophysics is the role of the extracellular matrix (ECM) and mechanical forces in modulating cell behavior. We first present an article by Druzhkova et al. [1] that examines how the ECM can influence cancer cells’ responses to chemotherapeutic agents. The authors visualized the distribution of doxorubicin in cells grown using 3D collagen-based models. They found that collagen, especially when remodeled by cancer cells, restricts drug delivery. Similarly, Jana et al. [2] developed novel nanofiber-based scaffolds of varying geometries to understand how 3D growth affects the biophysical properties of tunneling nanotubes (TNTs). TNTs are thought to play a role in cancer development by enabling communication between cancer cells and their environments, but most studies on them have been performed in 2D culture. The authors of [2] found that the 3D ECM geometry influenced the number, length, and organization of TNTs and that TNTs interact with the ECM in a suspended nanofiber network. To conclude this topic, Cambi and Ventre [3] published a review discussing the importance of collagen in the ECM and the types of ECM biomimetic scaffold that are available for in vitro research.

The following three articles take a biophysical approach to developing new cancer treatment strategies. Mathews et al. [4] sought to determine whether modifying the transmembrane potential of cancer cells could prevent proliferation. Using an automated whole-cell patch-clamp recording platform, they screened bioelectric drugs that target ion channels and change membrane potential, correlating changes in voltage with the promotion of differentiation and senescence. A study by Sobiepanek et al. [5] determined the outcomes of treating melanoma cancer cells with the endocannabinoid anandamide (AEA). A combination of biological, biomolecular, and biophysical methods revealed that AEA can reduce metastatic potential. The authors’ use of quartz crystal microbalance with dissipation monitoring (QCM-D) allowed them to detect real-time changes in the glycosylation profiles of cells treated with AEA. Shimolina et al. [6] used two-photon imaging and mass spectrometry to monitor chemotherapy-mediated changes in tumor cell membrane viscosity and lipid composition, respectively. They showed that treatment with the chemotherapeutic drug oxaliplatin increased the membrane viscosity and altered the phosphatidylserine and cholesterol content. Importantly, resistance to oxaliplatin was linked to the maintenance of lower viscosity in cells, suggesting that the prevention of this membrane adaptation may represent a new therapeutic target.

Dysregulation can occur at various levels, from single molecules to cell populations. This Special Issue concludes with two reviews that investigate the biophysical properties of proteins in the promotion of oncogenic signaling. A review from the Renz laboratory elaborates on the role of protein distribution and dynamics in differentiating normal cells from cancer cells, with a focus on comparing beta-catenin and CapG in gynecological cancers [7]. The role of quantitative live cell microscopy is highlighted here, as CapG is found to be uniformly distributed throughout the cell, but its nucleocytoplasmic shuttling is faster in cancer cells—a result that could not be uncovered using immunohistochemistry or genomic profiling. Nagy and colleagues conclude this Special Issue with a review of receptor tyrosine kinases (RTKs) in cancer, providing a thoughtful discussion of the structure–function relationships and lipid-mediated interactions that regulate RTK function, as well as an excellent overview of the biophysical measurements that have contributed to our mechanistic understanding of this topic [8].

The collection of articles in this Special Issue of *Cancers* contains exciting examples of how biophysical approaches can provide new insights into oncogenic processes. One of the key contributions of biophysical approaches is the ability to investigate intact and living cells at multiple spatiotemporal scales. Thus, the continued incorporation of biophysical perspectives and techniques into the field of cancer biology will undoubtedly advance our mechanistic understanding of oncogenesis and facilitate the development of novel therapeutic targets and combination therapies.
